# Accelerometry-Based Physical Activity and Affective Responses to Daily Stressors: An Analysis of the AAPECS Study

**DOI:** 10.1093/geroni/igab046.165

**Published:** 2021-12-17

**Authors:** Eli Puterman, Benjamin Hives, A Janet Tomiyama, Carissa Low, Geralyn Ruissen, Mark Beauchamp, Aidan Wright

**Affiliations:** 1 University of British Columbia, University of British Columbia, British Columbia, Canada; 2 University of British Columbia, Vancouver, British Columbia, Canada; 3 University of California Los Angeles, Los Angeles, California, United States; 4 University of Pittsburgh, Pittsburgh, Pennsylvania, United States

## Abstract

Evidence suggests that physical activity on a daily basis dampens the extent to which one experiences elevations in negative affect in response to daily stressors. Yet, these studies primarily relied solely on end-of-day recall of stressors and negative affect, and self-reported physical activity. More intensive assessments throughout the day and accelerometry-based physical activity measurements are required to answer whether any type of body movement (e.g. light, moderate, vigorous) reconfigures the end-of-day recall of the intensity of the affective experience of a stressor or, rather, mitigates the actual experience of a stressor in real-time. This presentation will summarize results addressing this question using data from the University of Pittsburgh’s Assessment of Personality, Ecological Context, and Stress (AAPECS) study. AAPECS includes172 participants who wore accelerometers to assess movement-based activities and completed ecological momentary assessment 6 times daily for 14 days, with additional ‘bursts’ of affective assessments following reported stressors at any time.

